# Network pharmacology-based investigation of potential targets of mulberry twig acting on polycystic ovary syndrome

**DOI:** 10.1097/MD.0000000000046163

**Published:** 2025-11-21

**Authors:** Qing Jiang, Danfeng Pu, Yun Hu, Ruoyan Wu, Chenjie Jiang, Shiqin Yuan, Xiaowei Zhu, Lan Xu

**Affiliations:** a Department of Endocrinology, Nanjing Medical University, The Affiliated Wuxi People’s Hospital of Nanjing Medical University, Wuxi, China; b Department of Endocrinology, The Affiliated Geriatric Hospital of Nanjing Medical University, Nanjing, Jiangsu Province, China.

**Keywords:** molecular docking, mulberry twig, network pharmacology, polycystic ovary syndrome

## Abstract

**Background:**

Polycystic ovary syndrome (PCOS) is a polygenic multifactorial systemic inflammatory autoimmune disease. Mulberry twig (MT) has pharmacological activities such as anti-inflammatory, hypoglycemic, anti-oxidant, and insulin resistance. Our study aimed to understand whether MT can affect PCOS and to assess its potential targets.

**Methods:**

PCOS targets were searched using the OMIM, TTD, and GeneCards databases. The active components and corresponding protein targets of MT were searched in the Traditional Chinese Medicine Systems Pharmacology (TCMSP) database, and the compound-target network was constructed using Cytoscape 3.8.0. The intersection of the compound and disease targets was obtained, and the coincidence target was imported into the STRING database to construct a protein–protein interaction (PPI) network. Gene ontology (GO) and Kyoto Encyclopedia of Genes and Genomes (KEGG) enrichment analyses were performed on these targets. Finally, molecular docking methods were used to confirm the high affinity between the bioactive molecules of MT and their targets in PCOS.

**Results:**

TCMSP database results showed that the 3 active components of MT acted against PCOS. The PPI network and core target analysis suggested that AKT1, TNF, and CASP3 are key targets of PCOS. KEGG analysis showed that MT treatment in PCOS mainly involved fluid shear stress and atherosclerosis. GO analysis showed that positive regulation of the apoptotic process, caveola, and enzyme binding play an important role in MT in PCOS. Molecular docking methods confirmed the high affinity between the bioactive molecules of MT and their targets in PCOS.

**Conclusion:**

MT may serve as a promising therapeutic candidate for PCOS, as verified by the network pharmacology approach based on data mining and molecular docking methods. However, further in vivo and in vitro experiments are needed.

## 1. Introduction

Polycystic ovary syndrome (PCOS) affects approximately 5% to 15% of women worldwide^[1]^ and is the most common endocrine and metabolic disease among women of reproductive age.^[2]^ PCOS is a clinical syndrome that includes hyperandrogenism, oligo-anovulation, and polycystic ovarian morphology,^[3]^ which is frequently associated with insulin resistance, abdominal adiposity, obesity, metabolic disorders, and cardiovascular risk factors. Numerous variables contribute to PCOS,^[4]^ such as genetic or lifestyle factors. PCOS can be caused by thyroid dysfunction, hyperprolactinemia, androgen secretion tumors, Cushing syndrome, and congenital adrenal hyperplasia.

Currently, metformin is the primary medication used to treat PCOS.^[5]^ However, the side effects of metformin include lactic acidosis, severe drowsiness, cold skin, muscle pain, nausea, vomiting, and diarrhea.^[6]^ Steroid hormone treatment is another strategy for treating PCOS. Aromatase inhibitors are used to induce ovulation, spironolactone is administered to treat androgenism, N-acetylcysteine is administered to treat insulin resistance, and clomiphene citrate is administered to treat anovulatory infertility.^[7,8]^ The long-term use of these hormone-altering drugs can lead to a variety of complications, including obesity, cancer, and psychiatric problems. In conclusion, all the above-mentioned therapies are effective, but not entirely, to a certain extent. However, these therapeutic modalities have side effects.

Owing to its fewer side effects and satisfactory efficacy, traditional Chinese medicine has been successfully used for the treatment of PCOS.^[9]^ Mulberry twigs (MT) are plants that mostly grow in East Asian nations, such as China, and have been used as a traditional medicine to treat inflammation,^[10]^ obesity,^[11]^ and diabetes.^[12]^ The main components of MT include alkaloids, flavonoids, polysaccharides, coumarins, amino acids, and organic acids.^[13]^ Its active compounds show some health benefits, such as cholesterol reduction and attenuation of acute colitis. Previous studies have demonstrated diverse pharmacological effects of MT, such as the regulation of blood glucose concentration, insulin secretion, and insulin resistance status.^[14]^ MTs have become a potential drug for the treatment of PCOS. Although MT has been widely used clinically, its therapeutic effect in PCOS needs to be investigated.

**Figure 1.**
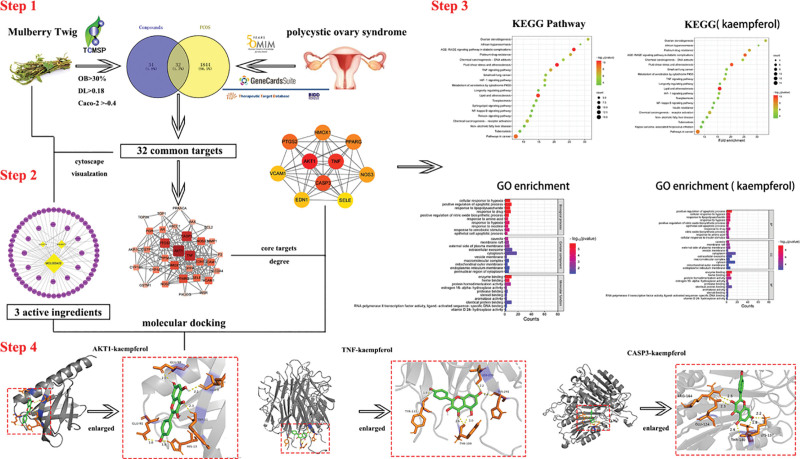
Workflow of the study design. After screening the targets related to PCOS and MT, the overlapping targets were subjected to KEGG pathway enrichment analysis and GO functional enrichment analysis, followed by verification via molecular docking.Caco-2 = the absorption and transport of drugs in intestinal epithelial cells, DL = drug-likeness, GO = gene ontology, KEGG = Kyoto Encyclopedia of Genes and Genomes, MT= mulberry twig, OB = oral bioavailability.

Network pharmacology is a unique, promising, and cost-effective method for identifying bioactive components, forecasting drug action targets, and studying drug action processes from the perspective of biological network balance^[15,16]^ based on public databases and publicly available data. In our study, network pharmacology was used to analyze the active ingredients, potential targets, and pathways of MT for the treatment of PCOS. The molecular docking experiments further verified these results. The study design and workflow are shown in Figure 1.

## 2. Materials and methods

### 2.1. Screening the active ingredients and targets of MT

Active components of MT were retrieved from the Traditional Chinese Medicine Systems Pharmacology Database (TCMSP, https://www.tcmsp-e.com/#/home).^[17]^ The term oral bioavailability (OB) describes the pace and volume at which a medicine is absorbed into the bloodstream. Drug-like (DL) features describe the nature of a drug when it belongs to a particular functional category or has the same or similar physical attributes. The human intestinal cell line Caco-2 is a useful tool for studying drug absorption and transport in intestinal epithelial cells. As previously reported,^[18]^ OB > 30%, DL > 0.18, and Caco-2 permeability > -0.4 were used to further screen the compounds with higher activity. The target proteins corresponding to each molecule were obtained from the TCMSP Database and converted to a unified gene name using the protein database UniProt^[19]^ (http://www.uniprot.org/uploadlists/).

### 2.2. Construction of active compound-target network

Cytoscape is a network biology visualization and analysis application that visualizes molecular connections and biological processes (BP).^[20,21]^ For visualization, potential active components and matching targets of MT were imported into Cytoscape 3.8.0, and a network of MT components and targets was constructed. Each component or target in the compound-target network is represented as a node, and the connection between each component and target is shown as a connecting line.

### 2.3. Collection of predicted targets of PCOS

Using the keywords “polycystic ovary syndrome” and “Homo sapiens,” PCOS-related targets were found in the OMIM (https://omim.org/),^[22]^ GeneCards(https://www.genecards.org/),^[23]^ and therapeutic target databases (https://db.idrblab.net/ttd/). By combining the targets in these 3 databases, duplicate targets were removed, and the final targets were the MT targets we collected. A Venn diagram was constructed by mapping the MT drug targets to the disease targets of PCOS.

### 2.4. Construction of PPI network and analysis of core targets

To learn more about the protein-protein interaction (PPI) network, the targets from the cross set were imported into the STRING database (https://string-db.org/) to learn more about the PPI network. The biological screening condition was set to “Homo sapiens,” and the minimum required interaction score was medium confidence (0.400).^[24]^ PPI data were loaded into Cytoscape 3.8.0 for PPI network visualization and development. The top 10 scores of target proteins in network string interactions were output, which was ranked by the Maximal Clique Centrality and degree method using the Cytohubba plug-in.^[25]^ For each node in the network graph, Cytoscape can calculate additional characteristics such as degree, betweenness centrality (BC), and closeness centrality (CC).^[26]^ These parameters enable an in-depth analysis of the properties of the nodes in the interaction network. We then selected BC and CC target nodes with PPI network median values above the corresponding median values and predicted 10 possible core targets.

### 2.5. GO enrichment and KEGG pathway analysis

The Kyoto Encyclopedia of Genes and Genomes (KEGG) is an encyclopedia of genes and genomes, assigning functional significance to genes and genomes at the molecular level and beyond.^[27]^ The life sciences have made extensive use of the GO, which offers structured, calculable knowledge about the function of genes and gene products.^[28]^ Therefore, for GO and KEGG functional enrichment analyses, the DAVID database^[29]^ (https://davidbioinformatics.nih.gov/) was used. Subsequently, GO and KEGG data were uploaded to the bioinformatics (http://www.bioinformatics.com.cn/) platform for visual analysis.

### 2.6. Molecular docking

Molecular docking, a popular computer-based structural method for drug development, may predict ligand-target interactions at the molecular level.^[30]^ To determine whether the active components identified by network pharmacology might bind to the primary targets, we performed molecular docking. The 3-dimensional (3D) structure of the target protein was downloaded from the PDB Database (https://www.rcsb.org/). Simultaneously, the structure of the drug small molecule was downloaded from the TCMSP Database. They were later dehydrated, hydrogenated, and stored in *pdbqt* format using AutoDock. The protein was designated as the receptor and the drug was set as the ligand. To ensure that the target protein was entirely covered by the docking box, we imported the receptor and ligand *pdbqt* structures into AutoDock and set the target protein as the center of the grid. Finally, PyMOL software was used to visualize the outcomes. In this study, a trustworthy binding was defined as one with a binding free energy of less than −1.2 kcal/mol.^[31]^

## 3. Results

### 3.1. Candidate compounds and targets of MT in PCOS

**Figure 2.**
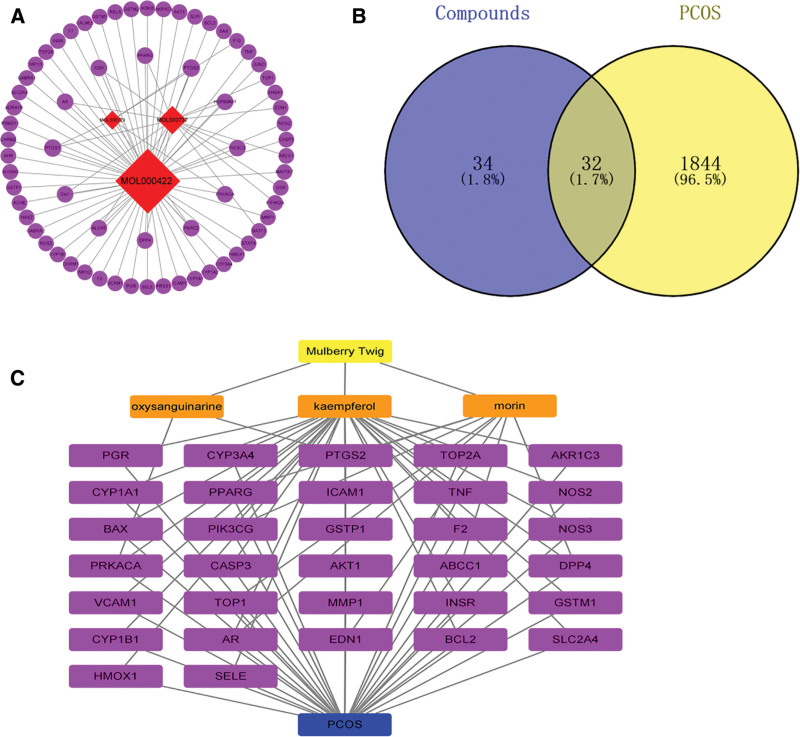
Active ingredients and targets of MT. (A) Ingredient-target network of MT. The rhombus represented the active ingredient and the circle represented the target gene. The bigger the rhombu, the more genes it targeted. (MOL000422: kaempferol; MOL000729: oxysanguinarine; MOL000737: morin). (B) Venn diagram of the target of MT and the target of PCOS. (C) The network of PCOS-common targets-ingredients-MT. MT= mulberry twig, PCOS = polycystic ovary syndrome.

First, 3 active ingredients of MT (Table 1) and 66 related targets (Table S1, Supplemental Digital Content, https://links.lww.com/MD/Q748) were selected from the TCMSP database according to OB, DL, and Caco-2. Based on the multi-component and multi-target characteristics of traditional Chinese medicine, Cytoscape 3.8.0 software was used to construct the MT component target network. As shown in Figure 2A, there were 69 nodes and 80 edges. A total of 1876 targets of PCOS (Table S2, Supplemental Digital Content, https://links.lww.com/MD/Q748) were obtained from the 3 above-mentioned disease databases. The relevant targets for PCOS and MT were mapped on a Venn diagram, and 32 overlapping targets were obtained (Fig. 2B). Based on this, a network of disease-common targets-ingredients-herbs was constructed. Figure 2C shows that kaempferol, the key active ingredient in MT, had 29 common targets with PCOS, morin was the second key active ingredient with 8 common targets, and oxysanguinarine had 2 common targets. Of these, PTGS2 is related to kaempferol, morin, oxysanguinarine, and PCOS. Therefore, targeting PTGS2 may be one of the main mechanisms of action of MT in the treatment of PCOS.

**Table 1:**
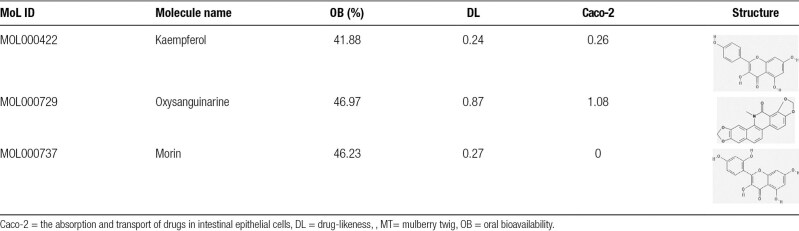
Screening results of active ingredients in MT.

### 3.2. PPI network construction and analysis

**Figure 3.**
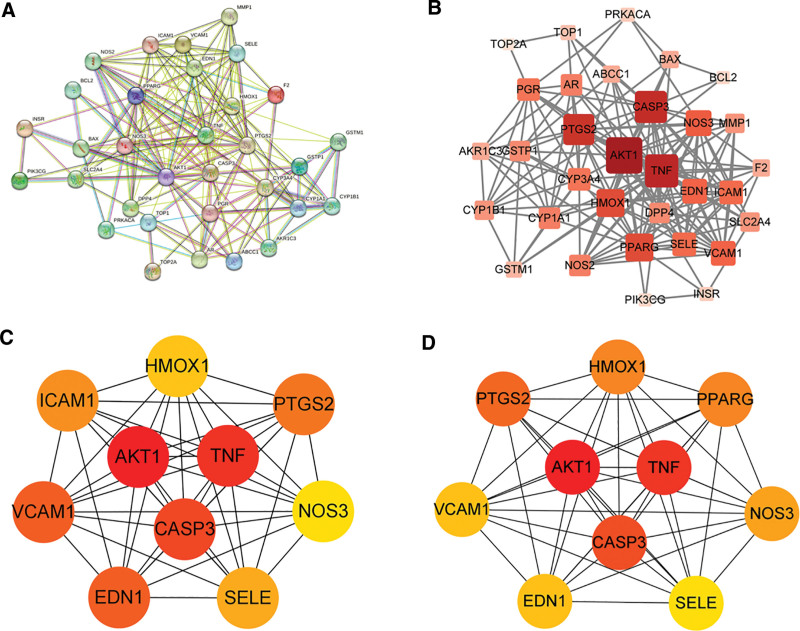
The construction and analysis of PPI network. (A and B) PPI network of potential targets of MT in treatment of PCOS. (C) The network of 10 hub genes obtained by MCC. (D) The network of 10 hub genes obtained by degree. MCC = maximal clique centrality, MT= mulberry twig, PCOS = polycystic ovary syndrome, PPI = protein–protein interaction.

To investigate the mechanism of MT in PCOS, a PPI network was created by adding 32 overlapping targets to the STRING database. Cytoscape then received PPI network data, which were created using the STRING platform (Fig. 3A). To create a PPI network, common targets were visualized. The larger the node in the diagram, the more red the color, and the more likely it was to be a core target (Fig. 3B). As shown in Table 2, we filtered out the top 10 targets with the degree values of BC and CC. The top 10 core targets were further calculated in the Cytohubba plug-in using the MCC and degree algorithms (Fig. 3C and D). As AKT1, TNF, and CASP3 had the highest scores (scoring information is presented in Tables S3 and S4 [Supplemental Digital Content, https://links.lww.com/MD/Q748]), they may play a crucial role in the control of PCOS.

**Table 2:** Top 10 targets information of PPI network.

Name	Betweenness centrality	Closeness centrality	Degree
AKT1	0.178587721	0.837837838	25
TNF	0.096485766	0.775000000	22
CASP3	0.088149545	0.756097561	21
PTGS2	0.059163369	0.738095238	20
PPARG	0.043006874	0.673913043	17
HMOX1	0.036701471	0.673913043	17
NOS3	0.031867726	0.645833333	15
CYP3A4	0.031765552	0.620000000	12
PGR	0.062946558	0.620000000	12
AR	0.024045853	0.607843137	11

PPI = protein–protein interaction.

### 3.3. GO enrichment and KEGG pathway analysis

**Figure 4.**
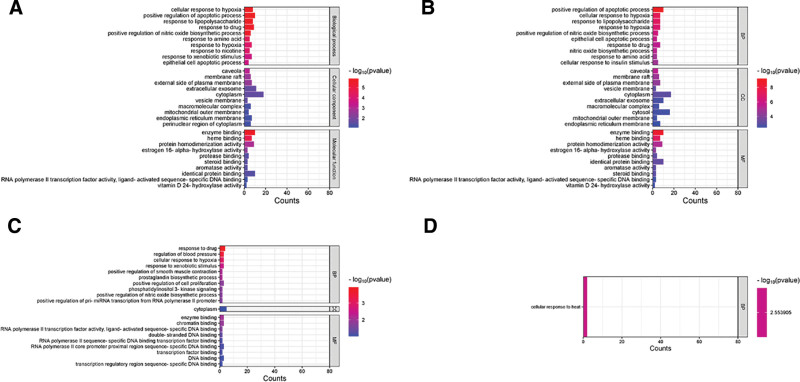
GO enrichment analysis. (A) The top 10 enriched BPs/CCs/MFs based on the adjusted *P*-value and ordered by Counts. (B) The top 10 GO terms of targets shared by kaemperol and PCOS. (C) The main GO terms of targets shared by morin and PCOS. (D) GO enrichment analysis of common targets of oxysanguinarine and PCOS. BP = biological process; CC = cellular components; GO = gene ontology, MF= molecular function, PCOS = polycystic ovary syndrome.

**Figure 5.**
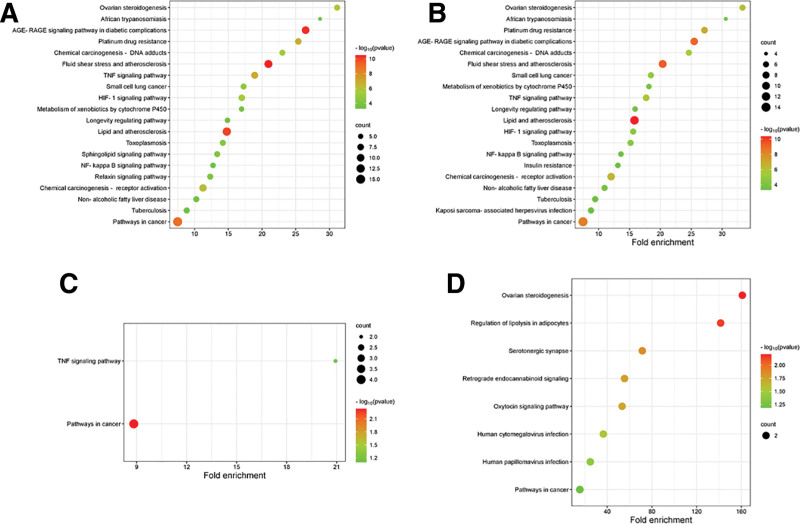
KEGG enrichment analysis. (A) The top 20 KEGG pathways based on the adjusted *P*-value and ordered by fold enrichment. (B) The top 20 pathways of kaemperol against PCOS. (C and D) Pathways of morin/oxysanguinarine against PCOS. KEGG = Kyoto Encyclopedia of Genes and Genomes, PCOS = polycystic ovary syndrome.

To elucidate the mechanism of action of MT in PCOS, 32 targets were subjected to GO and KEGG enrichment analyses. As shown in Figure 4A, 10 BP, cellular components (CC), and molecular functions (MF) were screened out in the GO enrichment analysis. The main BPs were the positive regulation of the apoptotic process (GO:0043065), cellular response to hypoxia (GO:0071456), response to lipopolysaccharide (GO:0032496), and positive regulation of the nitric oxide biosynthetic process (GO:0045429). In addition, the main CC was caveola (GO:0005901) and the main MF was enzyme binding (GO:0019899). We further analyzed the possible mechanisms of action of the active ingredients of MT for the improvement of PCOS. The shared targets of kaempferol and PCOS included 298 significant items (adjusted *P*-value < .05), of which 234 were BPs, 25 were CCs, and 39 were MFs. However, the common targets of morin and PCOS involved 37 significant items, and oxysanguinarine involved one BP (Fig. 4B–D). KEGG pathway analysis indicated that common targets were enriched in 89 pathways (Table S5, Supplemental Digital Content, https://links.lww.com/MD/Q748). The top 20 ranked pathways based on –log_10_ (*P*-value) and count are shown in Figure 5A. Among them, 12 pathways were correlated with human diseases, 4 with environmental information processing, 3 with organismal systems, and 1 with metabolism. The key pathways of kaempferol were lipid and atherosclerosis, fluid shear stress, atherosclerosis, and the AGE-RAGE signaling pathway in diabetic complications (Fig. 5B). The pathways of morin were cancer and TNF signaling pathways (Fig. 5C), while the pathways of oxysanguinarine were ovarian steroidogenesis and regulation of lipolysis in adipocytes (Fig. 5D) (details of KEGG enrichment are shown in Table S6 [Supplemental Digital Content, https://links.lww.com/MD/Q748]).

### 3.4. Molecular docking

**Figure 6.**
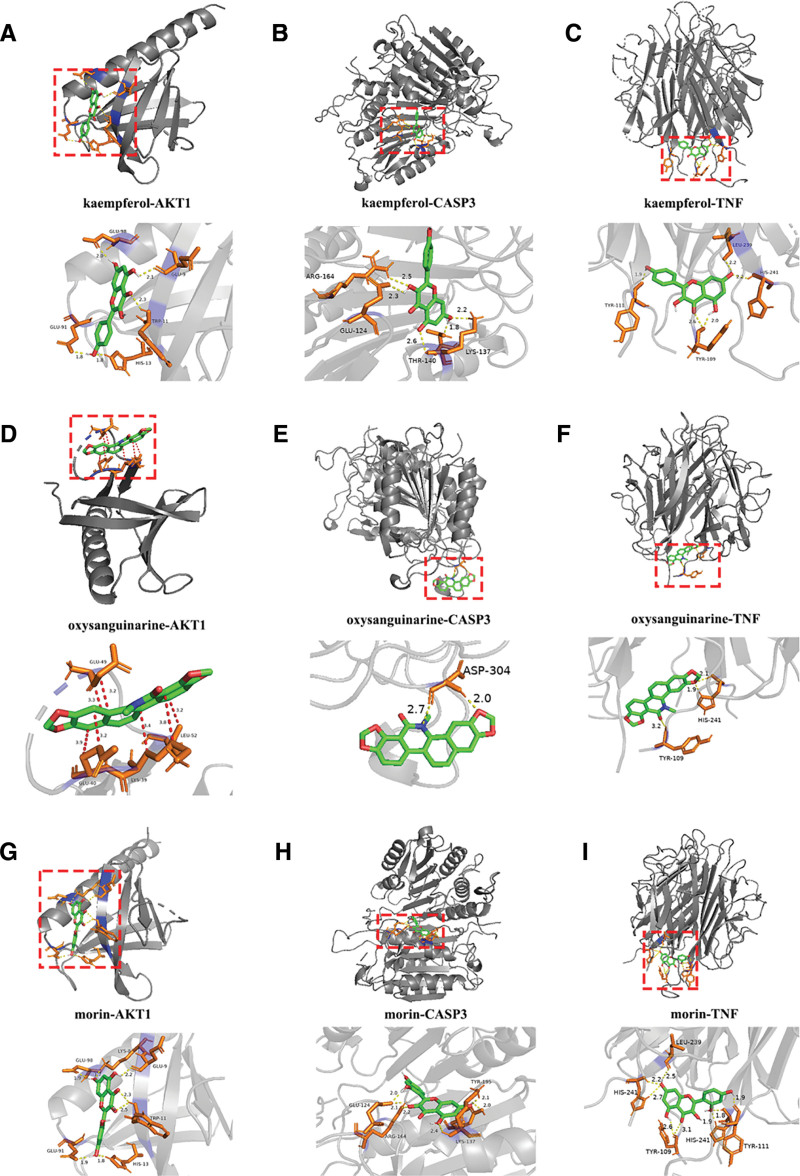
The results of molecular docking between ingredients and hub genes. (A–I) Interactions between the receptor and the ligand. The red dotted line, and yellow dotted line indicate that the interaction is hydrophobic and hydrogen bond respectively. The number nearby indicates the magnitude of the acting force. Caco-2 = the absorption and transport of drugs in intestinal epithelial cells, DL = drug-likeness, OB = oral bioavailability.

To test whether the ingredients bound effectively to their targets, we performed molecular docking of the 3 active ingredients of MT with the top 3 core genes AKT1 (PDB ID: 7MYX), TNF (PDB ID: 6U66), and CASP3 (PDB ID: 6X8I). The results showed that these components had a high affinity to the hub protein, where the free energy of binding affinity was less than −5 kcal/mol, as illustrated in Table 3. Among them, docking of TNF and oxysanguinarine had the highest binding energy (−8.88 kcal/mol), and docking of CASP3 and morin had the lowest binding energy (−5.59 kcal/mol). Figure 6 depicts the optimal docking of the receptor and ligand after visualization. These results demonstrated that kaempferol, morin, and oxysanguinarine could interact with AKT1/TNF/CASP3. The hydrogen bond interaction force, which is the primary force promoting molecule binding to the active site, is illustrated by the yellow lines. These active components may establish specific hydrogen bonds within certain regions of a target protein. Although morin and AKT1 did not establish any hydrogen bonds, our later research revealed that they interacted via hydrophobic forces. In summary, kaempferol, morin, and oxysanguinarinebound well to the 3 primary targets (AKT1, TNF, and CASP3). Based on this, we hypothesized that these substances might be crucial in the treatment of PCOS.

**Table 3:** Docking results of core target proteins and core active components.

Compound\affinity (kcal/mol)	AKT1 (7MYX)	TNF (6U66)	CASP3 (6X8I)
Kaempferol	−6.49	−7.52	−5.6
Oxysanguinarine	−6.55	−8.88	−7.55
Morin	−5.98	−7.55	−5.59

## 4. Discussion

The average rate of obesity in women with PCOS is approximately 49%.^[32]^ Additionally, they also exhibit higher serum concentrations of TNF and C-reactive protein and circulating levels of monocytes and lymphocytes, as well as inflammatory infiltration of ovarian tissue.^[33]^ Elevated insulin levels promote the apoptosis of ovarian granulosa cells, which in turn inhibits ovulation. In addition, insulin stimulates androgen and adrenal secretion.^[34]^ These results suggest that PCOS is associated with chronic low-grade inflammation, obesity, and insulin resistance.

As few medications are currently available to treat PCOS, the primary goal of this study was to identify whether MT could help in treating PCOS and to assess the underlying mechanisms. Using the TCMSP database, a total of 23 chemical components were collected, including kaempferol, morin, and oxysanguinarine, which could act on multiple targets in the network.

Kaempferol is a natural flavonoid widely distributed in the food, beverage, and plant kingdoms. Kaempferol has a significant inhibitory effect on cyclooxygenase,^[6]^ which is activated when physical, chemical, or mechanical damage occurs in any part of the body. Therefore, kaempferol inhibits cyclooxygenase and prevents the inflammatory processes. Kaempferol is also a good antioxidant that scavenges free radicals and inhibits lipid peroxidation.^[35]^ Studies have revealed that kaempferol decreases insulin resistance and controls lipid metabolism to reduce lipotoxicity. At the same time, kaempferol improves insulin signaling and restores the balance between glucose utilization and production, thereby improving glucose toxicity. In addition, kaempferol can restore the imbalance in autophagy to protect β-cells. Our molecular docking results demonstrated that kaempferol exhibits good binding ability to the core targets of PCOS (AKT1, TNF, and CASP3). As an active component of MT, kaempferol can alleviate PCOS symptoms by inhibiting inflammation, regulating lipid metabolism, and improving insulin resistance. In addition, another active ingredient of MT – morin – demonstrated powerful anti-inflammatory and antioxidant effects.^[36,37]^ Currently, there are few studies on the effects of oxysanguinarine.

We gathered PCOS targets from the OMIM, GeneCards, and therapeutic target databases. We found that PCOS and MT share 32 targets. These targets have been identified as potential targets of MT for the treatment of PCOS. To explore the core targets, we built a PPI network expressing PPIs. The PPI network results showed that AKT1, TNF, CASP3, PTGS2, HMOX1, and NOS3 may be the core targets, especially AKT1, TNF, and CASP3. Studies have reported that the disease state of PCOS appears to be associated with low expression of AKT in ovarian tissue.^[38]^ In our study, AKT was ranked first regardless of the algorithm used. Therefore, targeting AKT could be a potential therapeutic intervention for PCOS. We confirmed the interaction between MT and AKT using molecular docking. These results suggest that AKT is a key target of MT in PCOS treatment. In addition, we found that PTGS2, although ranked fourth, is a common target of kaempferol, oxysanguinarine, morin, and PCOS. Yu et al found that PTGS2 expression was increased in PCOS mice and was significantly decreased by treatment with β-sitosterol.^[39]^ Further in vitro and in vivo experiments are required to verify whether MT can improve PCOS symptoms by targeting PTGS2.

To explore the mechanism of MT in PCOS treatment, we conducted GO and KEGG enrichment analyses. GO results showed that the target genes were mainly enriched in BP, such as positive regulation of the apoptotic process. The total serum oxidative status, oxidative stress index, and granule cell apoptosis rate were significantly increased in the follicular fluid of PCOS patients.^[40]^ Pregnant rats with PCOS exhibit reduced apoptosis in the uterus and increased necrosis in the placenta.^[41]^ KEGG enrichment analysis revealed that MT involves multiple signaling pathways in the treatment of PCOS, mainly including fluid shear stress, atherosclerosis, and the AGE-RAGE signaling pathway in diabetic complications. Polycystic ovary syndrome is associated with an increased risk of cardiovascular diseases, including coronary heart disease and stroke.^[3]^ Azhary et al. found that hyperandrogenism in PCOS increases the expression of receptor for AGEs (RAGE) and accumulation of advanced glycation end products (AGEs) in the ovary by activating endoplasmic reticulum stress.^[42]^ This suggests that targeting these 2 signaling pathways could be a new direction for the treatment of PCOS.

The current investigation has certain limitations. We explored the role of MT in PCOS using web-based pharmacology and molecular docking. However, current web-based information technology needs to be further improved, and the accuracy and timeliness of the data in the database need to be scientifically validated.^[43]^ Unidentified and undocumented compounds or targets may not have been included in our analysis. The 3 bioactive components identified in our study are not fully representative of MT and need to be further validated using pharmacodynamic and molecular biology experiments, in addition to in vivo and in vitro testing.

## 5. Conclusion

In summary, we identified the active components of MT and its potential targets in the control of PCOS. The therapeutic effect of MT on PCOS was achieved by modulating lipid metabolism and insulin levels, and by improving the inflammatory microenvironment. Although some of the results reported in this work still require experimental validation, we believe that they will provide new insights for drug development in PCOS.

## Acknowledgments

The authors would like to thank the authors of the references section.

## Author contributions

**Conceptualization:** Lan Xu.

**Data curation:** Qing Jiang, Danfeng Pu, Ruyan Wu, Chenjie Jiang.

**Formal analysis:** Ruyan Wu, Shiqin Yuan.

**Funding acquisition:** Lan Xu.

**Methodology:** Qing Jiang.

**Software:** Qing Jiang.

**Supervision:** Xiaowei Zhu.

**Validation:** Lan Xu.

**Writing – original draft:** Qing Jiang.

**Writing – review & editing:** Qing Jiang, Yun Hu.

## Supplementary Material


